# Audit to Assess the Availability of Electrocardiogram (ECG) Machines on the Inpatient Units at Leeds and York Partnership Foundation Trust (LYPFT)

**DOI:** 10.1192/bjo.2025.10698

**Published:** 2025-06-20

**Authors:** Sabah Yasin, Abhijit Chakrabarti

**Affiliations:** Leeds and York Partnership NHS Foundation Trust, Leeds, United Kingdom

## Abstract

**Aims:** Establish the scale of the problem by collecting data on how frequently electrocardiogram (ECG) machines are not available, and the time doctors are spending searching for them.

Develop strategies for better monitoring and maintenance of available machines.

**Methods:** Initial data was collected from resident doctors within Leeds and York Partnership Foundation Trust (LYPFT) regarding incidents where ECG machines were not available over a period of 3 months beginning 01/08/2024 and ending 01/11/2024.

Data collection was facilitated by sending emails to resident doctors three times over the course of data collection. A reminder message was also sent out to the Resident Doctors WhatsApp group. Reports were received via email.

The data was collated and recorded on an Excel spreadsheet by SY.

Following data collection, statistical analysis was done on data received. This was via qualitative analysis such as calculation of the mean, median, mode; and through qualitative analysis via thematic analysis.

Due to the concerns surrounding early reports received and the implications for patient safety, concerns were escalated in the Trust senior leadership meetings and more ECG machines were sourced during the audit period.

Results were discussed at the Physical Health Team monthly meeting, to consider potential for improvements.

**Results:** A total of 28 reports were received over the three-month period, with the majority in August prior to escalating to senior leadership. Doctors spent a total of 490 minutes searching for a machine, with the mode and median being 10 minutes, and the longest being 120 minutes. 4 incidents reported of ECGs not being done. 89% of ECGs were required for routine monitoring, with 11% being due to chest pain. 46% of incidents were due to a missing machine, and 54% were due to a faulty machine. Faults were due to a paper fault, broken leads, missing clips, no charging cables, or the machine itself not working.

**Conclusion:** There are clearly significant issues with the availability of ECG machines across the inpatient facilities within the trust, leading to potentially significant delays both for routine and urgent ECGs. Issues highlighted within the trust meetings suggested that faulty machines were often not reported or fixed. To address this, it has been agreed to develop instructional flowcharts to streamline the escalation process and to implement this within the trust over the coming months.

